# A Case of Subacute Combined Degeneration of the Spinal Cord with Infective Endocarditis

**DOI:** 10.1155/2015/327046

**Published:** 2015-09-07

**Authors:** Xiao-Jiang Huang, Jia He, Wen-sheng Qu, Dai-Shi Tian

**Affiliations:** Department of Neurology, Tongji Hospital, Tongji Medical College, Huazhong University of Science and Technology, Wuhan, Hubei 430030, China

## Abstract

*Background*. Subacute combined degeneration (SCD) is a rare cause of demyelination of the dorsal and lateral columns of spinal cord and is a neurogenic complication due to cobalamin deficiency. Anemia of chronic disease (ACD) occurs in patients with acute or chronic immune activation, including infective endocarditis. It remains to be elucidated whether ACD patients are more sensitive to suffer from SCD. Little cases about SCD patients accompanied with ACD have been reported till now. Here we reported a 36-year-old man with SCD with a medical history of mitral inadequacy over 20 years, who was admitted and transported from another hospital to our hospital due to an 8-month history of gait disturbance, lower limb weakness and paresthesia, and loss of proprioception. Significant laboratory results and echocardiography suggest iron deficiency anemia and infective endocarditis (IE). The SCD diagnosis was confirmed by MRI, which showed selective demyelination in the dorsal and lateral columns of spinal cord. In conclusion, the ACD patients may suffer from SCD, which can be diagnosed by 3 Tesla magnetic resonance imaging.

## 1. Background

Myelopathy secondary to cobalamin deficiency is defined as subacute combined degeneration (SCD), involving progressive degeneration of the spinal cord, optic nerve, and peripheral nerves. And it is manifested by lower limb weakness, loss of proprioception, and so forth. In the past century, cobalamin deficiency is usually considered to be associated with pernicious anaemia [1], an autoimmune disease caused by autoantibodies against parietal cells, achlorhydria. The nitrous oxide exposure during anaesthesia is a rare cause of acute vitamin B12 inactivation [2]. New mechanisms are responsible for the pathogenesis of SCD: the neuropathological lesions in the totally gastrectomized rats are not only due to mere vitamin withdrawal but also due to the overproduction of the myelinolytic tumor necrosis factor- (TNF-) *α* and the reduced synthesis of the two neurotrophic agents, epidermal growth factor (EGF) and interleukin-6. This deregulation of the balance between TNF-*α* and EGF synthesis induced by cobalamin deficiency has been verified in the sera of patients with pernicious anemia and in the cerebrospinal fluid of SCD patients [3]. Anemia of chronic disease (ACD) is the second most prevalent after anemia caused by iron deficiency and occurs in patients with acute or chronic immune activation, including infective endocarditis [4].

## 2. Case Presentation

A 36-year-old man was admitted to our hospital due to an 8-month history of gait disturbance, lower limb weakness, loss of proprioception, described like lower limbs being “step on cotton,” and urinary incontinence. He had a medical history of a mitral inadequacy over 20 years, with no preexisting diabetes mellitus, alcohol addiction, or gastrointestinal symptoms. Physical examination on presentation revealed emaciated, low-grade fever, and normal blood pressure. Auscultation of heart and lungs revealed no major abnormalities. The neurological examination showed weakness (3/5) in his lower limbs and hyperactive deep tendon reflexes in the lower extremities. Babinski's sign, Romberg's sign, and Lhermitte's sign were positive. Vibration and joint position sense examination were evaluated as decreased. There was complete absence of vibration sense in the lower limbs and proprioception and tactile sensation were impaired to the level of the knees but these were normal in the upper limbs. Pain and temperature sensation were normal.

**Figure 1:**
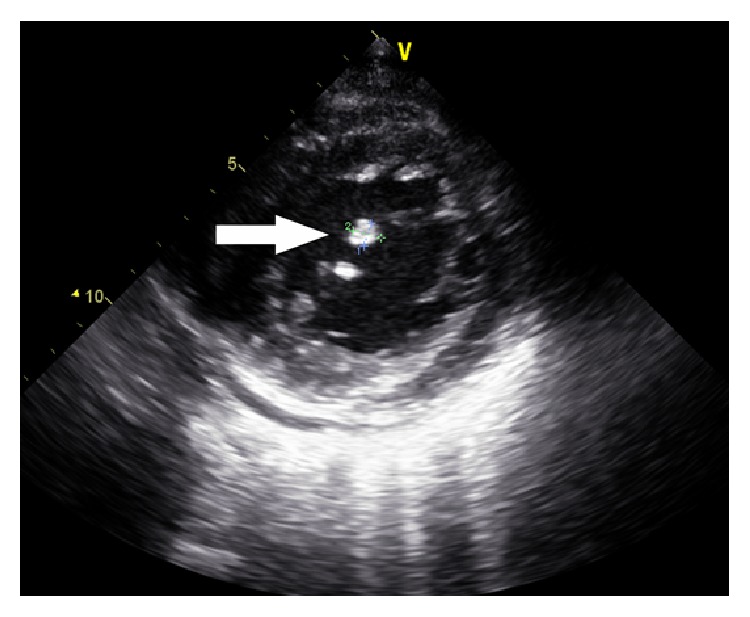
Echocardiography of the patient's showed vegetation on the mitral valve leaflet (7.67 mm*∗*6.14 mm), mitral valve prolapse and moderate regurgitation, mild degree backstreaming of tricuspid valve, and left ventricular enlargement.

Electroneurography and electromyography findings of his legs have shown neurogenic damage, suggesting damaging of peripheral nerves. Evoked potentials performed showed abnormalities in both upper and lower limb somatosensory evoked potentials. Significant laboratory results included relative neutrophilia, normal BNP (827 pg/mL), antistreptolysin O (ASO) (<25 iu/mL), coagulation function, homocysteine (HCY) (26.4 mmol/L), serum copper level (16 *μ*mol/L), and blood culture (negative), decreased red cell count (2.83*∗*10^12^/L), hemoglobin (83 g/L), serum iron level (SI) (3.2 *μ*mol/L), total iron binding capacity (TIBC) (29.9 *μ*mol/L), and the reticulocyte count (1.2%), and increased high-sensitivity C-reactive protein (CRP) (60 mg/L), elevated erythrocyte sedimentation rate (ESR) (78 mm/h), rheumatoid factors (63 iu/mL), microalbuminuria (U-mAlb) (31.5 mg/L), serum ferritin level (902 ng/mL), and urine occult blood (3+) (urine red blood cells 121 per/*μ*L, homogeneity in 65%, and heterogeneity 35%), suggesting iron-deficient anemia and possible infective endocarditis (IE). Serum vitamin B12 level was markedly reduced (72 pg/mL) with decreased level of folate (1.31 ng/mL). The diagnosis of IE was confirmed by his echocardiography which showed vegetation on the mitral valve leaflet, mitral valve prolapse and moderate regurgitation, left ventricular enlargement (Figure 1). Color Doppler Ultrasonography of double kidney was normal.

**Figure 2:**
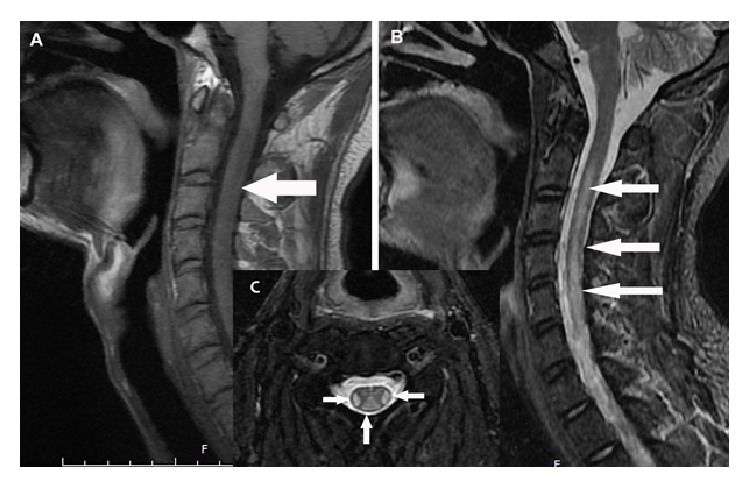
(A) T1-weighted MRI scans showing the dorsal spinal cord with hypointensity involving the posterior and lateral columns before treatment. (B) T2-weighted MRI scans showing the dorsal spinal cord with hyperintensity involving the posterior and lateral columns before treatment. (C) Transverse T2-weighted MRI scan of the cervical spinal cord at the C3 level demonstrating bilateral symmetric signal intensity within the dorsal and lateral columns (inverted V sign) before treatment.

The MRI findings of his brain were nonspecific, but the cervical vertebral MRI demonstrated T1 hypointense and T2 hyperintense signal in the paramedian dorsal and lateral cervical cord extending from C3 to C6 (Figure 2(A-B)). The axial MRI of C3 region clearly showed the typical “inverted V sign” [5], which was due to a characteristic involvement of the dorsal columns (Figure 2(C)). His MR imaging was suggestive of selective demyelination in the dorsal and lateral spinal cord.

The patient was treated intramuscularly with cyanocobalamin 1000 *μ*g daily for the first week and 1000 *μ*g/week for further 6 months. The patient had slow but progressive neurological improvement in his clinical symptoms and correction of anemia. The cardiac surgeon suggested that he could have a surgery of mitral valve replacement, which could help correct the anemia.

## 3. Discussion

Subacute combined degeneration (SCD) is a clinical entity associated with especially long-standing pernicious anemia that affects the different columns of the spinal cord and the peripheral nerves [3]. Infective endocarditis is considered as one underlying cause of anemia of chronic disease, and patients with mitral valve prolapse and valve regurgitation have a 10–100-fold increased risk of infective endocarditis. Anemia of chronic disease occurs in patients with acute or chronic immune activation [4]. The pathophysiological mechanisms underlying anemia of chronic disease are cytokines such as TNF-*α*, interleukin-1, interleukin-6, and interleukin-10 produced by immune system reaction to the invasion of microorganisms, which induce ferritin expression and stimulate the storage and retention of iron within macrophages and inhibit duodenal absorption of iron and the differentiation and proliferation of erythroid progenitor cells, and so forth, which lead to iron deficiency anemia. The key aspect in the pathogenesis of human and experimental SCD cobalamin is a reference molecule of the balance between EGF, IL-6, and TNF-*α* production in the CNS. Recently it has been demonstrated that TNF-*α* levels are abnormally high and EGF levels are abnormally low in the CSF of SCD patients [6]. Increased TNF-*α* as a putative neuron-damaging molecule in the CNS is certain to be necessary for the development of experimental SCD in the totally gastrectomized rats, which was supported by the fact that intracerebroventricular microinjections of agents antagonizing TNF-*α* production largely prevented SCD-like lesions in the spinal cord white matter of the totally gastrectomized rats [7]. Another thing is that the abnormal conditions affecting balance between IL-6 and TNF-*α* in the CNS may cause cobalamin deficiency, which leads to demyelination. This imbalance between serum EGF and TNF-*α* level can be corrected by cobalamin replacement therapy.

## 4. Conclusion

In this case, the patient has a long medical history of mitral inadequacy and an accompanying infective endocarditis, which might cause anemia of chronic disease and then lead to subacute combined degeneration.

## Abbreviations

IE: Infective endocarditis
ACD: Anemia of chronic disease
TNF: Tumor necrosis factor
SCD: Subacute combined degeneration.
